# Books on Swedish Treatment

**Published:** 1912-03-02

**Authors:** A. W. Macleod

**Affiliations:** Surgeon, R.N. (retired). St. Brelade's Bay Hotel, Jersey


*To the Editor of* The Hospital.

### BOOKS ON SWEDISH TREATMENT.

*To the Editor of* The Hospital.

Sir,—I should be obliged if you or any of your readers could let me know if any book has been lately published on the Swedish methods of physical culture both as applied to health and disease, more especially surgical injuries. I believe there is a ward at St. Thomas's Hospital now set apart for physical remedial treatment. I must apologise for troubling you.—Yours faithfully,

A. W. Macleod, Surgeon, R.N. (retired).

St. Brelade's Bay Hotel, Jersey, February 3.

[A good work is Ostrom's "Massage and the Swedish Movements" (price 3s. 9d. post free). Can be obtained from The Scientific Press, Ltd., 28 and 29 Southampton Street, Strand, W.C.—Ed. The Hospital.]

